# A Method for Similarity Search of Genomic Positional Expression Using CAGE

**DOI:** 10.1371/journal.pgen.0020044

**Published:** 2006-04-28

**Authors:** Shigeto Seno, Yoichi Takenaka, Chikatoshi Kai, Jun Kawai, Piero Carninci, Yoshihide Hayashizaki, Hideo Matsuda

**Affiliations:** 1 Department of Bioinformatic Engineering, Graduate School of Information Science and Technology, Osaka University, Osaka, Japan; 2 Genome Exploration Research Group (Genome Network Project Core Group), RIKEN Genomic Sciences Center, RIKEN Yokohama Institute, Yokohama, Japan; 3 Genome Science Laboratory, Discovery Research Institute, RIKEN Wako Institute, Wako, Japan; The Jackson Laboratory, US; MRC-Harwell, UK; NHGRI-NIH, US; Lawrence Livermore National Laboratory, US; The Jackson Laboratory, US

## Abstract

With the advancement of genome research, it is becoming clear that genes are not distributed on the genome in random order. Clusters of genes distributed at localized genome positions have been reported in several eukaryotes. Various correlations have been observed between the expressions of genes in adjacent or nearby positions along the chromosomes depending on tissue type and developmental stage. Moreover, in several cases, their transcripts, which control epigenetic transcription via processes such as transcriptional interference and genomic imprinting, occur in clusters. It is reasonable that genomic regions that have similar mechanisms show similar expression patterns and that the characteristics of expression in the same genomic regions differ depending on tissue type and developmental stage. In this study, we analyzed gene expression patterns using the cap analysis gene expression (CAGE) method for exploring systematic views of the mouse transcriptome. Counting the number of mapped CAGE tags for fixed-length regions allowed us to determine genomic expression levels. These expression levels were normalized, quantified, and converted into four types of descriptors, allowing the expression patterns along the genome to be represented by character strings. We analyzed them using dynamic programming in the same manner as for sequence analysis. We have developed a novel algorithm that provides a novel view of the genome from the perspective of genomic positional expression. In a similarity search of expression patterns across chromosomes and tissues, we found regions that had clusters of genes that showed expression patterns similar to each other depending on tissue type. Our results suggest the possibility that the regions that have sense–antisense transcription show similar expression patterns between forward and reverse strands.

## Synopsis

Through the advancement of genome research, it is becoming clear that genes are not distributed on the genome in random order. Clusters of genes distributed at localized genome positions have been reported in several eukaryotes. Various correlations have been observed between the expressions of genes in adjacent or nearby positions along the chromosomes depending on tissue type and developmental stage. It is reasonable that genomic regions that have similar mechanisms show similar expression patterns. In this study, the authors analyzed gene expression patterns using the computational algorithm of similarity search for exploring systematic views of the mouse transcriptome. They found regions that had clusters of highly expressed genes in certain tissue types whose expression patterns showed strong similarity to each other. This work aims to provide additional insight into genome-wide mechanisms of transcription.

## Introduction

Advancements in genomic research have provided evidence that genes are not randomly distributed in the genome. In prokaryotes, clusters of coexpressed genes are mainly due to the presence of operons. Genes distributed within the same operon are transcribed together and are thus coregulated. Positional clustering analysis of genes on the chromosome helps detect functionally coupled genes and takes advantage of the fact that functionally related genes frequently inhabit the same neighborhood of the chromosome.

In general, eukaryotes lack operons. In eukaryotes, except in the case of tandem duplication, genes appear to be transcribed individually and are thought to be scattered throughout the chromosomes without apparent organization according to function or positional expression. However, positional clustering has recently been reported in several eukaryotes, including Saccharomyces cerevisiae [1,2], Drosophila melanogaster [3,4], Caenorhabditis elegans [5–7], Mus musculus [8–12], and Homo sapiens [13–16]. Cho et al. [1] first showed clustering of coexpressed yeast genes on a genome-wide scale. Such clustering was also reported by Cohen et al. [2], who computationally analyzed whole-genome gene expression using the same dataset. Using chromosome correlation maps, which display the correlations between the expression patterns of genes on the same chromosome, they found that adjacent or nearby nonadjacent pairs of genes showed similar expression patterns regardless of their orientation in the yeast genome. In addition, they showed that genes with similar functions tend to be adjacent along the chromosomes. Spellman and Rubin [3] found that numerous clusters that span 10–30 physically adjacent genes share strikingly similar expression profiles in D. melanogaster. These clustered genes accounted for over 20% of the total analyzed genes. Furthermore, by mapping expressed sequence tags back to the *Drosophila* genome, Boutanaev et al. [4] observed that almost one-third of 1,661 testis-specific genes are clustered. Similarly, positional clustering of coexpressed genes has also been reported in C. elegans. Although operon and tandem duplication are major mechanisms for the observed coexpression of gene clusters in the worm, there are additional explanations for the presence of gene clusters. Clusters of highly coexpressed genes have also been identified in the human and other mammalian genomes. Through analysis of expressed sequence tags and serial analysis of gene expression tags, positional clustering of several genes has been shown. Moreover, Su et al. [12] designed a custom array that interrogates the expression of the vast majority of protein-coding human and mouse genes. They used this dataset to search for chromosomal regions of correlated transcription, which may indicate higher-order mechanisms of transcriptional regulation. These studies suggest that there are clusters of tissue-specific genes, and that gene clustering might be more frequent than initially thought.

In addition, with the sequencing and annotation of genomes and transcriptomes of several eukaryotes, the importance of noncoding RNA (ncRNA)—RNA molecules that are not translated into protein products—has become more evident. Growing evidence indicates that a subclass of ncRNA transcripts participates in the regulation of many cellular functions in eukaryotes such as transcription interference and genomic imprinting. The fact that expression of the *SRG1* ncRNA is required for repression of *SER3* was reported as an example of transcription interference [17]. MicroRNAs (miRNAs) are a class of ncRNAs that downregulate the expression of their mRNA targets [18–20]. The miRNAs are encoded as short inverted repeats in the genomes of invertebrates and vertebrates, and they are believed to control translation by binding to the sites of antisense complementarity in 3′ untranslated regions. These mechanisms of transcriptional control mediated by ncRNAs are hard to predict from genomic sequences alone.

Several transcripts that are known to control the processes of epigenetic transcription occur in a cluster. Also, some genes in adjacent or nearby positions and in forward and reverse strands along the chromosomes influence the transcription of each other. These facts indicate that genomic regions that have similar mechanisms may have related patterns of expression, while expression is different depending on tissue type and developmental stage within the same genomic region.

Currently, the FANTOM3 project [21] provides one of the largest available resources for the discovery of transcriptional mechanisms in mammals. In addition to having a large collection of novel protein-coding transcripts, the FANTOM3 transcript set contains many ncRNAs, disease genes, antisense transcripts, and the cap analysis gene expression (CAGE) library. CAGE [22] is based on preparation and sequencing of concatamers of DNA tags derived from the initial 20 nucleotides at the 5′ ends of mRNAs. CAGE allows high-throughput analysis of gene expression and offers all the advantages of serial analysis of gene expression and expressed sequence tag sequencing, including the detection of rare and novel transcripts. The frequency of CAGE tags correlates well with the results of other expression analyses [23].

Here we describe an approach for exploring transcriptional mechanisms using the CAGE database, which allows simultaneous detection of the expression levels of the entire genome without a priori knowledge of gene functions. Mapping the CAGE tags back to the genome and counting the tags for each fixed-length region allowed us to determine genomic expression levels. These expression levels were normalized, quantified, and converted into the descriptors denoted by one of four characters [24,25]. The expression patterns along the genome were then regarded as character strings that could be analyzed using dynamic programming similar to that employed for sequence analysis [26]. We profiled genomic positional expression in the mouse genome and found that regions that show similar expression patterns encode clusters of highly expressed genes depending on tissue type. Furthermore, we showed the possibility that regions that have sense–antisense transcription show similar expression patterns between forward and reverse strands.

## Results/Discussion

### Comprehensive Similarity Search across Chromosomes and Tissues

**Figure 1:**
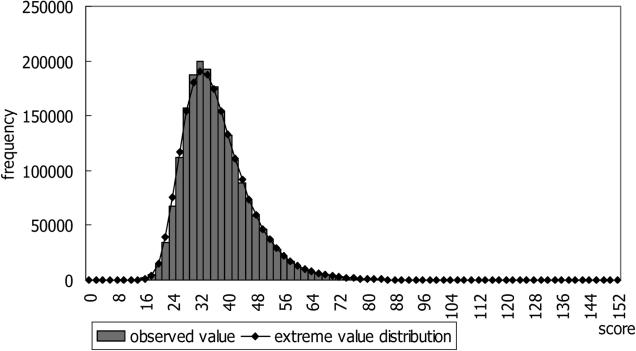
The Distribution of Observed Scores and the Extreme Value Distribution The bars show the distribution of observed scores, and the line indicates an extreme value distribution. The horizontal axis shows the score, and the vertical axis shows the frequency of the score.

Based on the idea that genomic regions that have similar mechanisms may have related patterns of expression depending on tissue type, we performed a similarity search of expression patterns in mouse across 21 chromosomes and 22 tissues using CAGE libraries. Counting the number of mapped CAGE tags for fixed-length regions allows determination of genomic expression levels. These expression levels were normalized, quantified, and converted into four types of descriptors, allowing the expression patterns along the genome to be represented by character strings. We analyzed these using dynamic programming in the same manner as for sequence analysis (details described in Materials and Methods). The distribution of the expression scores followed the extreme value distribution (Figure 1). Table 1 shows the top ten pairs of regions with high significance (more results are shown in Table S1).

**Table 1:**
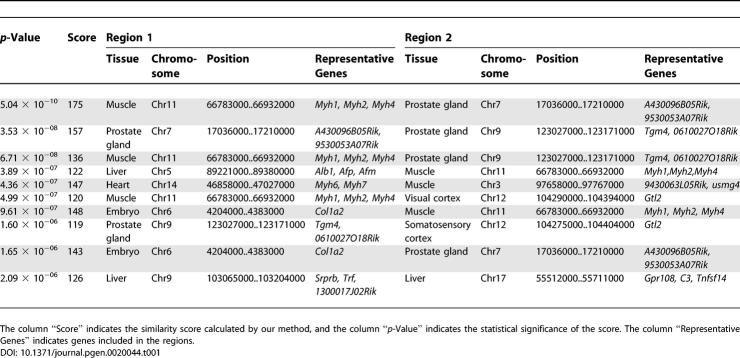
Top Ten Pairs of Regions with High Similarity of Expression Patterns

**Figure 2:**
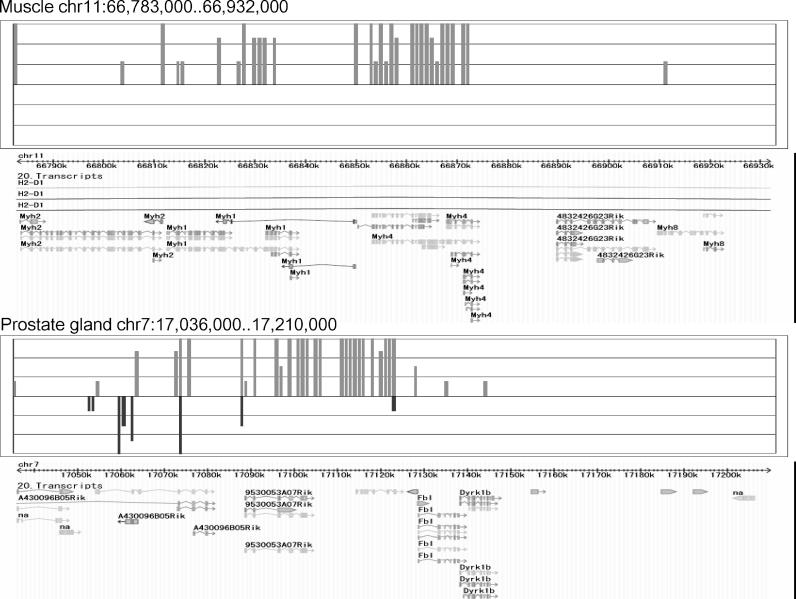
Genomic Expression Patterns for Chromosome 11 in Muscle and Chromosome 7 in Prostate Gland Expression pattern of Chromosome 11 in muscle, bases 66,783,001 to 66,932,000 (above), and Chromosome 7 in prostate gland, bases 17,036,001 to 17,210,000 (below). The upward and downward bars from N indicate the expression levels of the forward and reverse strands at the block, respectively. The genomic positional expression patterns of the two regions showed high similarity.

**Figure 3:**
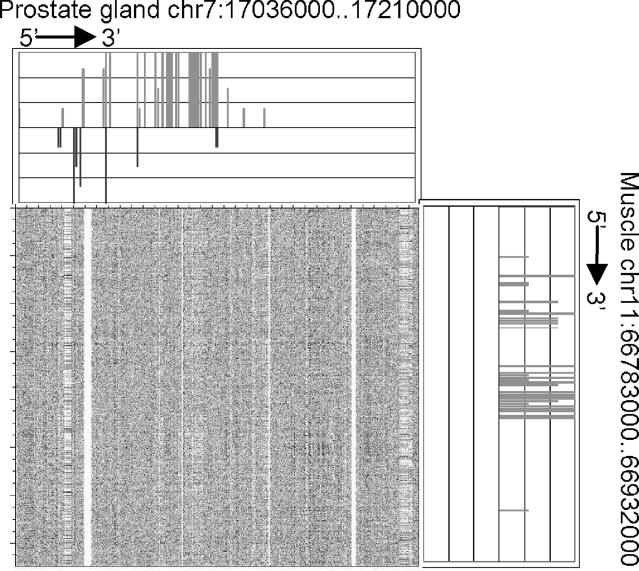
Genomic Expression Pattern and Sequence Similarity for Chromosome 11 in Muscle and Chromosome 7 in Prostate Gland Sequence similarity is shown for the same regions as in Figure 2. Expression patterns for Chromosome 11 in muscle and Chromosome 7 in prostate gland are shown at the right and at the top, respectively. The dot matrix shows the low sequence similarity of the two regions.

A region on Chromosome 11, bases 66,783,001 to 66,932,000, in the muscle and a region on Chromosome 7, bases 17,036,001 to 17,210,000, in the prostate gland showed the highest similarity in expression (score = 175, *p* < 5.04 × 10^−10^; Figure 2). The region on Chromosome 11 includes *Myh* genes that form a cluster on the genome and encode major structural proteins that participate in the function of skeletal muscles. The region on Chromosome 7 includes *A430096B05Rik* and *9530053A07Rik. A430096B05Rik* is Fc fragment of IgG binding protein, and *9530053A07Rik* is a hypothetical protein containing von Willebrand factor type D domain/EGF-like and trypsin inhibitor-like cysteine-rich domains. We used a dot matrix to evaluate the sequence similarity of these regions (Figure 3). The dot matrix provides a graphical method for comparing two sequences. One sequence is written horizontally across the top of the graph and the other is written vertically. Dots are placed within the graph at the intersections where the same nucleotide appears in both sequences. A series of diagonal lines in the graph indicate regions of alignment. The region on Chromosome 11 in the muscle and the region on Chromosome 7 in the prostate gland showed few sequence similarities (Figure 3), but there was high similarity of expression pattern.

A region on Chromosome 5, bases 89,221,001 to 89,380,000, in the liver showed high similarity in expression with a region on Chromosome 11, bases 66,783,001 to 66,932,000, in the muscle (score = 122, *p* < 3.89 × 10^−7^). The tandem arrangement of *Alb (albumin)* and *Afp (alpha-fetoprotein)* in the same transcriptional orientation has been well documented in mouse, rat, and human. *Afm (alpha- albumin/afamin),* the most recently identified member of the albumin gene cluster, is located immediately downstream and in the same transcriptional orientation as the *Afp* gene. These genes are expressed predominantly in the liver. We also observed similar expression patterns between the *Alb/Afp/Afm* cluster in liver and the *Myh* cluster in muscle (Figure S1). In contrast, these regions showed no apparent sequence similarities, same as in the case described above. Likewise for all of the pairs listed in Table 1, there were very few sequence similarities in spite of high expression similarities.

Moreover, a region on Chromosome 11, bases 66,665,001 to 67,165,000, in the muscle showed high similarity in expression with a region on Chromosome 6, bases 4,101,001 to 4,601,000, in the embryo (score = 148, *p* < 9.61 × 10^−7^). This region includes the *Col1a2* gene. The *Col1a2* gene, which encodes procollagen, type I, alpha 2, is one of the genes that provide instructions for making components of collagen. This gene is large (nearly 40,000 bases) and highly spliced. According to the Alternative Splicing Database (http://www.ebi.ac.uk/asd) [27], this gene has ten patterns of splicing. Because of its number of splice sites, the *Col1a2* gene showed an expression pattern similar to a gene cluster (Figure S2).

### Similarity Search between Forward Strand and Reverse Strand

We performed an expression similarity search between the forward and reverse strands of all transcriptional units (TUs) (39,593 TUs) in mouse. A TU was defined as the region or set of discontinuous regions in the genome from which all exons of a mature full-length mRNA are derived [28]. We selected three libraries that included more than 1,000,000 tags at the postnatal development stage: liver (1,420,891 tags), lung (1,129,858 tags), and macrophage (1,217,074 tags).

**Figure 4:**
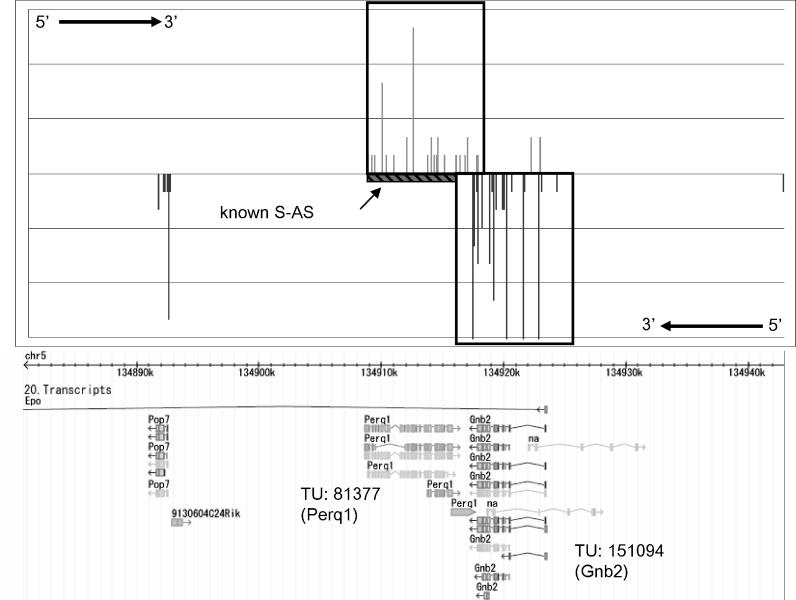
Genomic Expression Pattern for Chromosome 5, Bases 134,880,800 to 134,943,900, in Macrophage This region on Chromosome 5 shows similarity of expression pattern between the forward (TU 81377; *Perq1*) and reverse strands (TU 151094; *Gnb2*). The region from base 134,908,626 to base 134,893,818 (“known S-AS”) was reported by SADB as an overlapping region of sense–antisense transcription.

As an example of the results, Figure 4 shows the expression pattern of Chromosome 5, bases 134,880,800 to 134,943,900, which is the region that showed the highest similarity between the expression patterns of the forward and reverse strands in macrophage (*p* < 3.82 × 10^−9^). This region included TU 81377 in the forward strand and TU 151094 in the reverse strand. TU 81377 is *Perq1* (which encodes PERQ amino-acid-rich, with GYF domain 1) and TU 151094 is *Gnb2* (which encodes guanine nucleotide binding protein, beta 2). According to the Sense and Antisense Database (SADB; http://fantom31p.gsc.riken.jp/s_as) [29], known sense–antisense transcription exists between TU 81377 and TU 151094. More results for each tissue are provided in Tables S2 (liver), S3 (lung), and S4 (macrophage).

Table 2 shows the numbers of regions that showed high similarity between forward and reverse strands. The numbers of regions with known sense–antisense transcription are also indicated. This result demonstrates the possibility of inferring sense–antisense transcription from expression similarity searches between the forward and reverse strands.

**Table 2:**
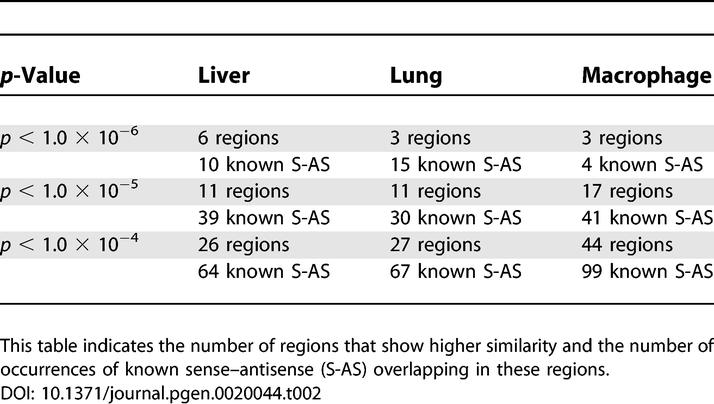
Results of Similarity Search between Forward and Reverse Strands

### Application for Genomic Positional Expression of miRNAs

We proposed a novel approach for providing a novel view of the genome. We searched for similarity of expression patterns across chromosomes and tissues and found regions that had clusters of genes that showed expression patterns similar to each other depending on tissue type. Furthermore, our results suggested that the regions that had sense–antisense transcription showed similar expression patterns between forward and reverse strands.

**Figure 5:**
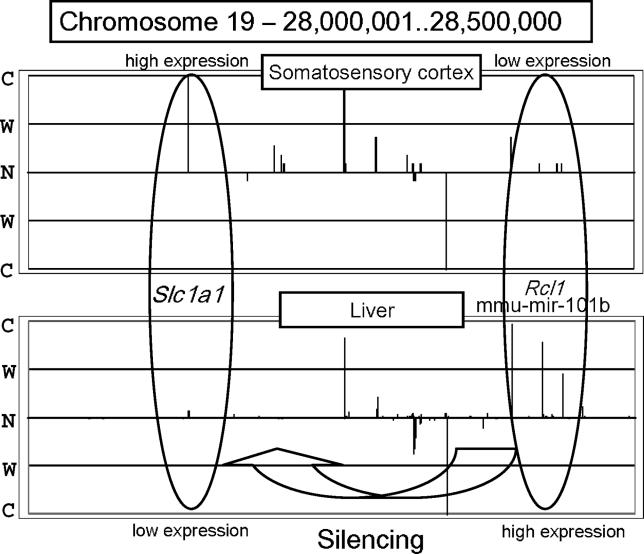
Comparison of the Expression Patterns between Somatosensory Cortex and Liver The upper panel shows the expression pattern on Chromosome 19, bases 28,000,001 to 28,500,000, in somatosensory cortex. The lower panel shows the expression pattern of the same region in liver. The two ellipses show the expression patterns of the regions encoding the *Slc1a1* gene and *mmu-mir-101b* miRNA (embedded in the intron of *Rcl1*). C, comparatively expressed; N, not expressed; S, strongly expressed; W, weakly expressed.

Our approach delivers valuable information and lends itself to various applications for the analysis of ncRNA expression. As an example of the application of our method, we observed the genomic positional expression of miRNAs. To characterize the expression patterns of regions encoding miRNAs, we collected the regions that had known miRNAs. The information on miRNAs was obtained from the Rfam database (http://www.sanger.ac.uk/Software/Rfam) [30]. We applied our method to 235 regions of 500,000 bases from 22 tissues that included known miRNAs. Those miRNAs that could not be confidently placed on a specific chromosome were excluded. We found that some of the regions exhibited interesting tendencies. Figure 5 shows the expression pattern for Chromosome 19, bases 28,000,001 to 28,500,000, in the somatosensory cortex and in the liver. According to TargetScan (http://genes.mit.edu/targetscan) [31], which combines thermodynamics-based modeling of RNA–RNA duplex interactions and comparative sequence analysis for predicting miRNA targets conserved in multiple genomes, *Slc1a1* appears to be a target of *mmu-mir-101b*. The miRNA *mmu-mir-101b* is embedded in the intron of *Rcl1*. *Slc1a1* encodes solute carrier family 1, member 1, and *Rcl1* encodes RNA terminal phosphate cyclase-like 1. In the somatosensory cortex, *Slc1a1* (left oval in Figure 5) was expressed strongly and *Rcl1* (right oval) was expressed weakly. This tendency was observed in cerebellum, visual cortex, and cerebral cortex. In contrast, *Slc1a1* was expressed weakly and *Rcl1* was expressed strongly in the liver, lung, embryo, and adipose tissue. This finding suggests the possibility that the region that includes *mmu-mir-101b* folds back upon the mRNA precursor, forming a stem loop so that the matured miRNA can silence the *Slc1a1* gene as a target.

### Additional Factors Affecting the Analysis

In conclusion, our method provided a way of quantifying the patterns of CAGE tag distribution, thus enabling extraction of higher-level gene expression patterns from the genome. It also provided a scoring function based on dynamic programming. In this study, we used a fixed and predefined length as block size. Because the results will vary depending on block size, the block size should be determined taking various factors into account, e.g., the size of the promoter (100–3,000 bases), the mRNA (1,000–20,000 bases), and the regions used to detect similarity (gene, gene cluster, or chromosome). However, the determination of optimal block size remains a difficult problem. A method for determining this parameter for more robustness is still under development.

The values used in the scoring matrix will also affect the results. The results calculated with the matrix used here (Table 3) had a tendency to detect similarities between regions that had a high density of genes with strong expression. To find weaker relationships, the scoring matrix should be modified in the same manner as other biological sequence analyses. With the matrix used here (Table 3), we could find regions that showed strong similarity to each other depending on tissue type. The results provided evidence for a nonrandom gene order and demonstrated the efficiency and potential of our method for further analysis of transcriptional mechanisms.

**Table 3:**
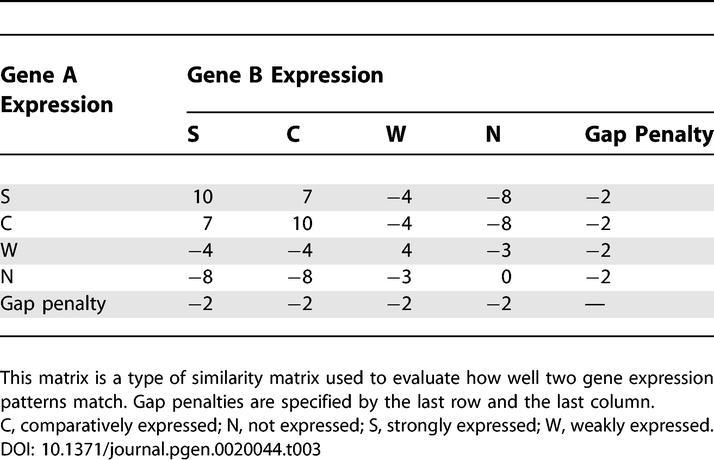
Scoring Matrix Used for Dynamic Programming

## Materials and Methods

### Mouse genome sequence and gene expression data.

In this study, we regarded the number of CAGE tags mapped to a region as a measure of the expression level for the region. We employed the data in the CAGE database (http://fantom31p.gsc.riken.jp/cage/mm5; M. musculus; Assembly version USCS-May-2004; Chromosomes 1–19, X, and Y) [21]. The dataset contains 6,895,911 CAGE tags uniquely mapped to the mouse genome from 22 tissues (except where the tissue type was undefined). Because the number of tags in each tissue is different (Table S5), it was difficult to directly compare the expression levels. To compare expression patterns across pairs of tissues or chromosomes, we converted the data to a string of descriptors as described below. Genomic expression patterns were quantified and normalized, and similarities between expression patterns were computed using dynamic programming similar to the Smith–Waterman algorithm [26].

### Composition of the frequency map.

To calculate the similarity between pairs of regions, we first constructed a frequency map for each chromosome and each tissue using the CAGE tags. We counted the number of tags mapped to each fixed-length block on the genome, and regarded the number of tags as the expression level for the block. In the analysis of similarity between chromosomes and tissues, the block size was set to 1,000 bases. We counted the number of CAGE tags that mapped to chromosome *c* in tissue *t* between bases 1 and 1,000, between bases 1,001 and 2,000, and so on. We defined the set of expression levels per block expressed in the forward strand on chromosome *c* in tissue *t,F_c_*(*t*) = {*F*_1,*c*_(*t*), *F*_2,*c*_(*t*), … 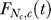 }, where *N_c_* is the number of blocks in chromosome *c,* and *F_i,c_*(*t*) is the number of tags expressed in *i*th block of chromosome *c* in tissue *t*. Likewise, the set of expression levels per block expressed in the reverse strand, *R_c_*(*t*), was defined as follows: *R_c_*(*t*) = {*R*_1,*c*_(*t*), *R*_2,*c*_(*t*), … 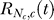 }. After counting all tags, we obtained the sequential expression levels along the chromosome. Figure S3 shows an overall view of expression levels on the mouse genome when the block size was set to 1,000,000 bases and all tissues were mixed. In the case of the similarity search between the forward and reverse strands, the block size was set to 100 bases.

### Conversion of data into descriptors.

To compare expression between two genomic regions, we converted the sequential expression levels in *F_i,c_*(*t*) and *R_i,c_*(*t*) to strings of descriptors, where a descriptor refers to how many tags were mapped in each block, and approximately represents the expression level. Binning the values is a good way to handle noise that may be introduced by experimental errors. Moreover, it allows us to focus on the more general tendencies of expression levels. Here, we defined four kinds of descriptors: S, strongly expressed; C, comparatively expressed; W, weakly expressed; and N, not expressed. Our method is an adaptation of the Event Method [24,25], which has been used for time-course expression profiling, e.g., in the mining of microarray data. We adapted the idea of representing expression levels by the use of symbol characters from time-course expression data to genomic positional expression data.

The conversion process involved the following steps. First, we calculated the standard deviation, *σ*(*t*), for each tissue per block. *Ē_c_*(*t*) represents the average of *F_c_*(*t*) and *R_c_*(*t*).


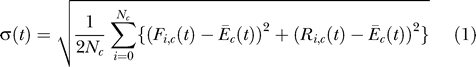


Next, we calculated scores to represent how many tags were expressed in the block compared to the whole.


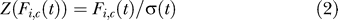



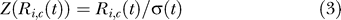


Thus, the series of expression levels *F_c_*(*t*) and *R_c_*(*t*) were transformed into descriptor sequences *M* such that


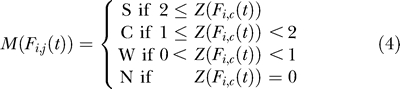



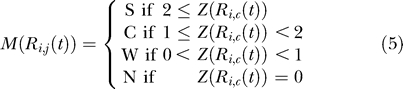


Finally, to calculate similarity, we picked out the sequential blocks along the chromosome. In the analysis of similarity across chromosomes and tissues, we set this length to more than 100 blocks. With a block size of 1,000 bases, we compared the expression patterns of regions more than 100,000 bases long. The whole mouse genome has more than 2 × 10^9^ bases. Even if the regions were picked out without overlap, there was a high computational cost. Therefore, we introduced a cut-off value to pick out regions. If the number of N (not expressed) descriptors included in the region was more than 80%, we regarded the region as less informative and rejected it. Figure S4 shows an example of a discarded region—liver, Chromosome 2, from block 26248 to block 26747 (bases 26,248,001 to 26,747,000)—and the descriptor strings of the forward and reverse strands of the region.

In the analysis of similarity between the forward strand and the reverse strand, we changed the length of descriptor sequences according to the size of each TU [27]. The number of TUs is 39,593 in mouse. The shortest TU is 29 bases and largest TU is over 2 million bases.

### Calculation of similarity of genomic expression.

Once the descriptor strings were obtained, we needed to determine whether the order of the descriptors indicated possible relationships by finding the best match between strings. We used an approach similar to that used in biological sequence alignment. Given the descriptor strings, we can efficiently determine their best alignment according to a suitably defined scoring function using a Smith–Waterman algorithm for local sequence alignment [26].


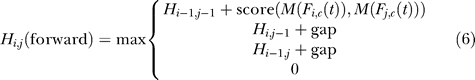



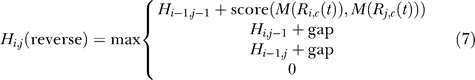


The Smith–Waterman alignment algorithm uses the scoring system to sort the sequence pairs. We set up the scoring matrix according to the idea of the Event Method [24,25]. As shown in Table 3, the matrix is a type of similarity matrix used to evaluate how well two gene expression patterns match. Gap penalties are specified by the last row and the last column. We calculated the similarity of the forward and reverse strands of the two regions individually, and then defined the similarity score between the regions as their sum.

For the similarity analysis between the forward strand and the reverse strand, we used the following mathematical formulation:


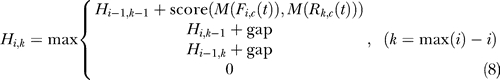


### Calculation of the significance of similarity scores.

The scores of our method followed the extreme value distribution, same as the scores of random sequence alignment. Figure 1 shows the distribution of scores observed using our method and the extreme value distribution. The equation of the extreme value distribution is


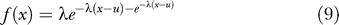


where *u* is the mode, highest point, or characteristic value of the distribution, and λ is the decay or scale parameter. There is an important relationship between *u* and λ and the mean and standard deviation of a set of extreme values. We calculated *u* and λ from means and standard deviations using the method of moments.

The probability that the score *S* will be less than value *x, P*(*S* < *x*), is obtained by calculating the area under the curve by integration of Equation 9, giving


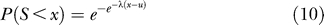


The probability of *S* ≥ *x* is thus


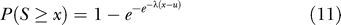


We regarded this probability as the statistical significance of the similarity score.

## Supporting Information

Figure S1 Genomic Expression Patterns for Chromosome 5 in Liver and Chromosome 11 in Muscle Expression pattern of Chromosome 5 in liver, bases 89,221,001 to 89,380,000 (above), and Chromosome 11 in muscle, bases 66,783,001 to 66,932,000 (below). The upward and downward bars from N indicate the expression levels of the forward and reverse strands at the block, respectively.
C, comparatively expressed; N, not expressed; S, strongly expressed; W, weakly expressed.
(605 KB PDF)

Figure S2 Genomic Expression Patterns for Chromosome 6 in Embryo and Chromosome 11 in Muscle Expression pattern of Chromosome 6 in embryo, bases 4,204,001 to 4,383,000 (above), and Chromosome 11 in muscle, bases 66,783,001 to 66,932,000 (below). The upward and downward bars from N indicate the expression levels of the forward and reverse strands at the block, respectively.
C, comparatively expressed; N, not expressed; S, strongly expressed; W, weakly expressed.
(652 KB PDF)

Figure S3 Overall View of Expression Levels for the Mouse Genome per 1,000,000 Bases The expression levels for each chromosome are indicated with a color code. High expression levels in the forward strand are shown in light red and low expression levels, are shown in dark red. High levels in the reverse strand are shown in light green, and low levels are shown in dark green. The blocks where very few or no tags were mapped are shown in black. The numbers at the left of the figure indicate the chromosome number.
(756 KB PDF)

Figure S4 An Example of Part of the Expression Pattern for Chromosome 2 in the Liver (A) Genomic expression pattern on Chromosome 2, bases 26,248,001 to 26,747,000, in liver. This figure includes 500 blocks from 26248 to 26747. The upward and downward bars from zero indicate the expression levels of the forward and reverse strands at the block, respectively. S (strongly expressed), C (comparatively expressed), W (weakly expressed), and N (not expressed) indicate the scale with the standard deviation σ(liver). The part of the panel shows information about the transcripts of this region.
(B) Converted descriptor string of the expression pattern of the forward strand.
(C) Converted descriptor string of the expression pattern of the reverse strand.
(96 KB PDF)

Table S1 Results of the Comprehensive Similarity Search: Pairs of Regions with Expression Pattern Similarity Significant at *p <* 0.001 The column “Score” indicates the similarity score calculated by our method, and the column “*p*-value” indicates the statistical significance of the score. Tissue type (“Tissue”), chromosome number (“Chr”), and chromosomal position (“Position”) are also indicated.
(67 KB XLS)

Table S2 Result of Similarity Search between Forward and Reverse Strands in Liver This table lists the regions that showed similar expression patterns between the forward and reverse strands in liver (*p* < 0.001). Statistical significances are indicated in the column “*p*-value.” Chromosomal positions for the forward and reverse strands are indicated. Information from the corresponding SADB entry is shown on the right. The column “Type” indicates the type of overlapping: C, convergent, tail-to-tail overlapping; D, divergent, head-to-head overlapping; F, full, fully overlapping.
(49 KB XLS)

Table S3 Result of Similarity Search between Forward and Reverse Strands in Lung This table lists the regions that showed similar expression patterns between the forward and reverse strands in lung (*p* < 0.001). Statistical significances are indicated in the column “*p*-value.” Chromosomal positions for the forward and reverse strands are indicated. Information from the corresponding SADB entry is shown on the right.
(53 KB XLS)

Table S4 Result of Similarity Search between Forward and Reverse Strands in Macrophage This table lists the regions that showed similar expression patterns between the forward and reverse strands in macrophage (*p* < 0.001). Statistical significances are indicated in the column “*p*-value.” Chromosomal positions for the forward and reverse strands are indicated. Information from the corresponding SADB entry is shown on the right.
(60 KB XLS)

Table S5 Number of CAGE Tags Mapped to Each Tissue The dataset contains 6,895,911 CAGE tags and 22 tissues. Each row indicates the number of tags in the corresponding tissues.
(81 KB PDF)

### Accession Numbers

The Entrez Gene (http://www.ncbi.nlm.nih.gov/entrez/query.fcgi?db=gene) GeneIDs for the genes discussed in this paper are *0610027O18Rik* (66079), *1300017J02Rik* (71775), *9430063L05Rik* (229622), *9530053A07Rik* (319482), *A430096B05Rik* (215384), *Afm* (280662), *Afp* (11576), *Alb* (11657), *C3* (12266), *Col1a2* (12843), *Gnb2* (14693), *Gtl2* (17263), *Myh2* (17882), *Myh4* (17884), *Myh6* (17888), *Perq1* (57330), *Rcl1* (59028), *Slc1a1* (20510), *Srprb* (20818), and *Tnfsf14* (50930).

## Abbreviations

CAGE: cap analysis gene expression
miRNA: micro RNA
ncRNA: noncoding RNA
SADB: Sense and Antisense Database
TU: transcriptional unit
