# Supplementary figures and images for: A Method for Similarity Search of Genomic Positional Expression Using CAGE
Source: PLoS Genet. 2006 Apr 28;2(4):e44. doi: 10.1371/journal.pgen.0020044 (PMC1449887; doi:10.1371/journal.pgen.0020044)

Liver chr5:89,221,001..89,380,000

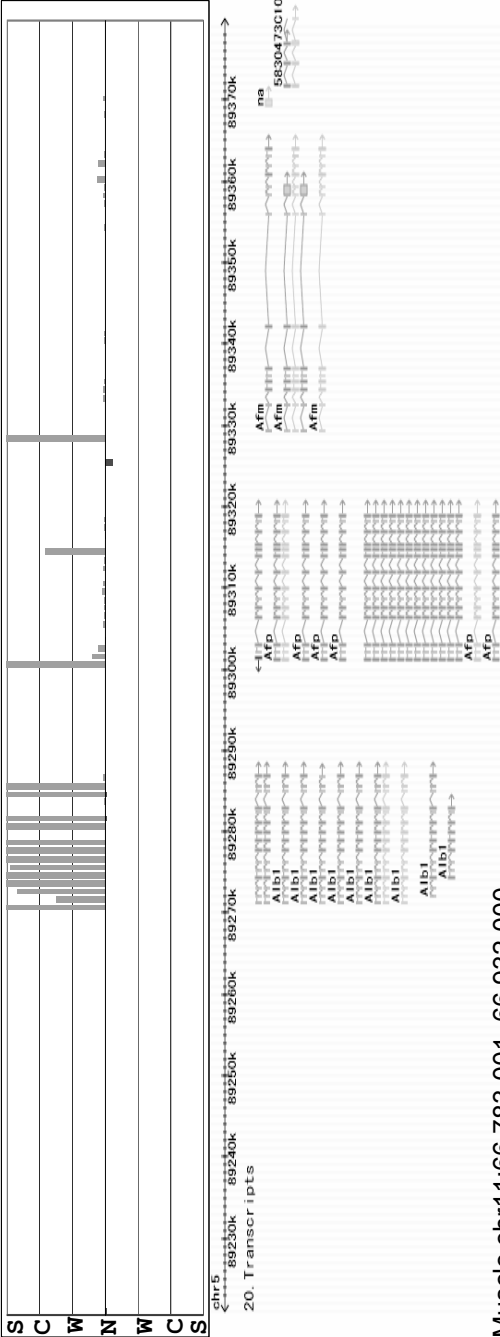

Muscle chr11:66,783,001..66,932,000

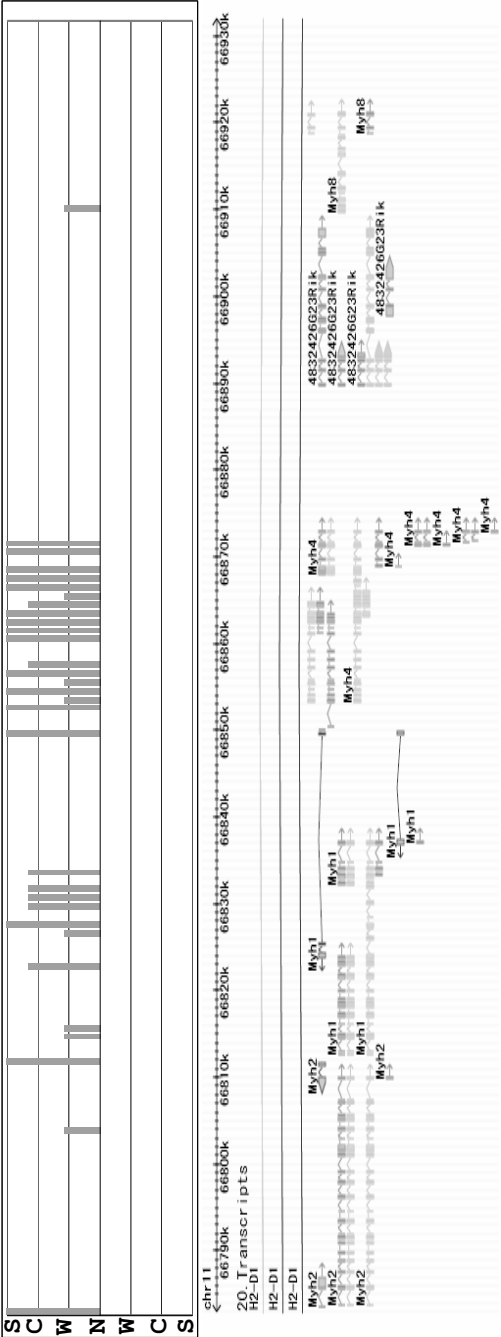

Supplement: Figure S1 — Expression pattern of Chromosome 5 in liver, bases 89,221,001 to 89,380,000 (above), and Chromosome 11 in muscle, bases 66,783,001 to 66,932,000 (below). The upward and downward bars from N indicate the expression levels of the forward and reverse strands at the block, respectively. C, comparatively expressed; N, not expressed; S, strongly expressed; W, weakly expressed. (605 KB PDF) [file pgen.0020044.sg001.pdf]

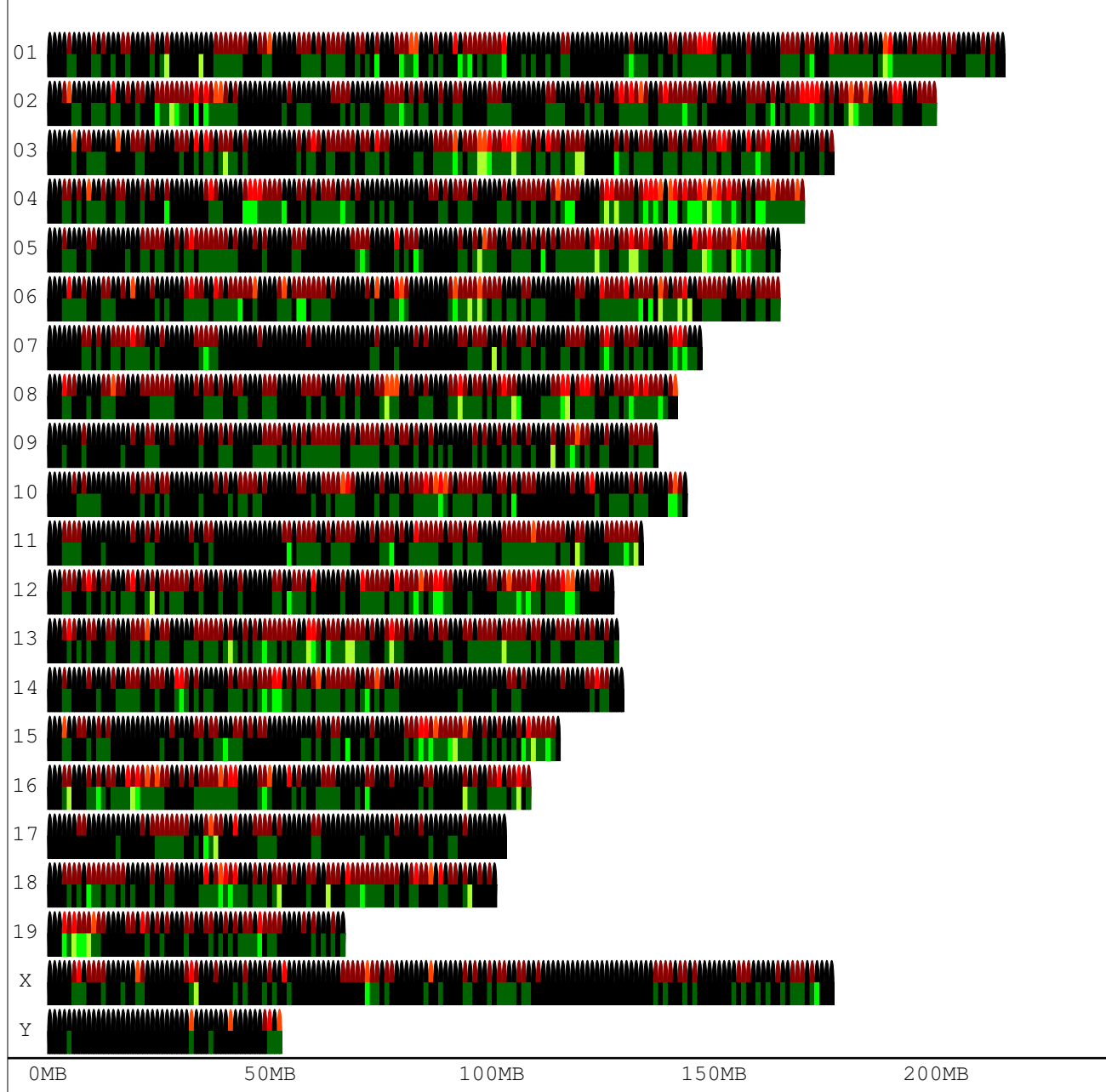

Supplement: Figure S3 — The expression levels for each chromosome are indicated with a color code. High expression levels in the forward strand are shown in light red and low expression levels, are shown in dark red. High levels in the reverse strand are shown in light green, and low levels are shown in dark green. The blocks where very few or no tags were mapped are shown in black. The numbers at the left of the figure indicate the chromosome number. (756 KB PDF) [file pgen.0020044.sg003.pdf]

**A.**

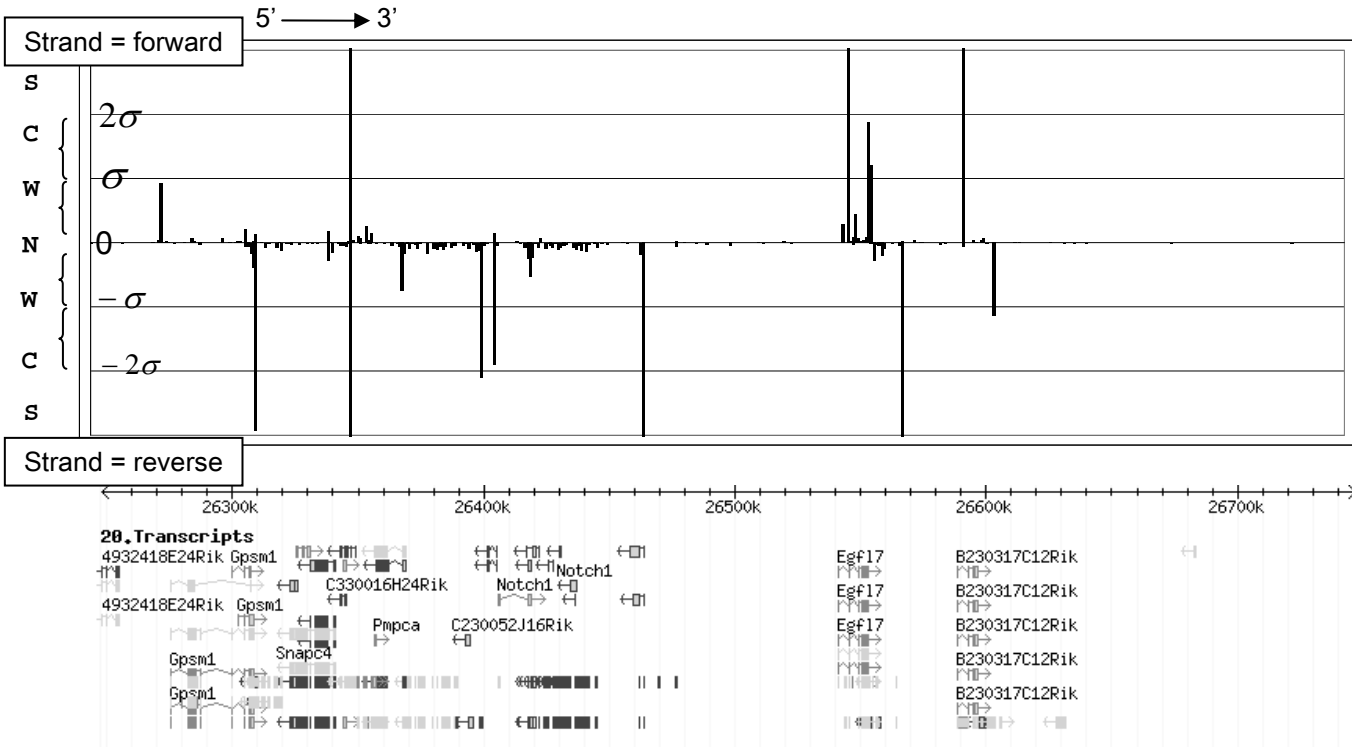

**B.**

[illegible]

**C.**

[illegible]

Supplement: Figure S4 — (A) Genomic expression pattern on Chromosome 2, bases 26,248,001 to 26,747,000, in liver. This figure includes 500 blocks from 26248 to 26747. The upward and downward bars from zero indicate the expression levels of the forward and reverse strands at the block, respectively. S (strongly expressed), C (comparatively expressed), W (weakly expressed), and N (not expressed) indicate the scale with the standard deviation σ(liver). The part of the panel shows information about the transcripts of this region. (B) Converted descriptor string of the expression pattern of the forward strand. (C) Converted descriptor string of the expression pattern of the reverse strand. (96 KB PDF) [file pgen.0020044.sg004.pdf]
