# Supplementary material for: A Method for Similarity Search of Genomic Positional Expression Using CAGE
Source: PLoS Genet. 2006 Apr 28;2(4):e44. doi: 10.1371/journal.pgen.0020044 (PMC1449887; doi:10.1371/journal.pgen.0020044)
Supplement: Table S5 — The dataset contains 6,895,911 CAGE tags and 22 tissues. Each row indicates the number of tags in the corresponding tissues. (81 KB PDF) [file pgen.0020044.st005.pdf]

Adipose	237,434
Amnion	381
Brain	122,603
Cerebellum	211,541
Cerebral cortex	17,510
Diencephalon	43,044
Embryo	1,077,740
Eye	1,246
Heart	34,743
Hippocampus	2,728
Liver	2,130,614
Lung	1,129,877
Macrophage	1,231,138
Mammary gland	1,294
Medulla oblongata	3,193
Muscle	42,294
Placenta	249
Prostate gland	57,057
Somatosensory cortex	190,762
Striatal primordia	39,693
Testis	109,150
Visual cortex	211,620
Sum	6,895,911
