# Supplementary material for: A Method for Similarity Search of Genomic Positional Expression Using CAGE
Source: PLoS Genet. 2006 Apr 28;2(4):e44. doi: 10.1371/journal.pgen.0020044 (PMC1449887; doi:10.1371/journal.pgen.0020044)
Supplement: Figure S2 — Expression pattern of Chromosome 6 in embryo, bases 4,204,001 to 4,383,000 (above), and Chromosome 11 in muscle, bases 66,783,001 to 66,932,000 (below). The upward and downward bars from N indicate the expression levels of the forward and reverse strands at the block, respectively. C, comparatively expressed; N, not expressed; S, strongly expressed; W, weakly expressed. (652 KB PDF) [file pgen.0020044.sg002.pdf]

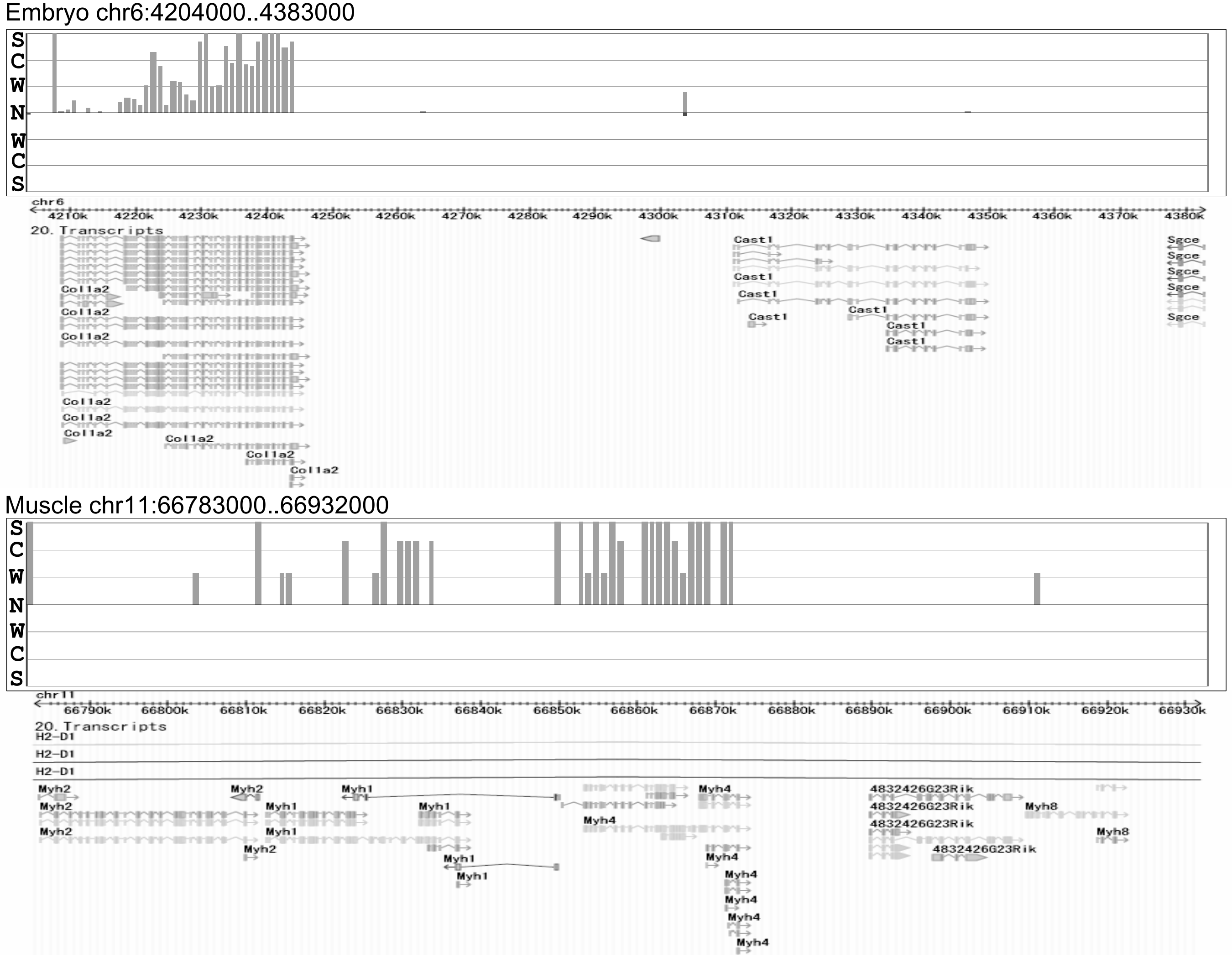
Embryo chr6:4204000..4383000
S
C
W
N
W
C
S
chr6
4210k
4220k
4230k
4240k
4250k
4260k
4270k
4280k
4290k
4300k
4310k
4320k
4330k
4340k
4350k
4360k
4370k
4380k
20. Transcripts
Col1a2
Cast1
Sgce
Muscle chr11:66783000..66932000
chr11
66790k
66800k
66810k
66820k
66830k
66840k
66850k
66860k
66870k
66880k
66890k
66900k
66910k
66920k
66930k
H2-D1
Myh2
Myh1
Myh4
4832426G23Rik
Myh8
